# Intestinal bacteria and colorectal cancer: etiology and treatment

**DOI:** 10.1080/19490976.2023.2185028

**Published:** 2023-03-16

**Authors:** Michael W. Dougherty, Christian Jobin

**Affiliations:** a Department of Medicine, University of Florida College of Medicine, Gainesville, FL, USA; b Department of Infectious Diseases and Immunology, University of Florida College of Medicine, Gainesville, FL, USA; c Department of Anatomy and Cell Biology, University of Florida College of Medicine, Gainesville, FL, USA

**Keywords:** Colorectal cancer, microbiome, genotoxin, inflammation, probiotics, immunotherapy

## Abstract

The etiology of colorectal cancer (CRC) is influenced by bacterial communities that colonize the gastrointestinal tract. These microorganisms derive essential nutrients from indigestible dietary or host-derived compounds and activate molecular signaling pathways necessary for normal tissue and immune function. Associative and mechanistic studies have identified bacterial species whose presence may increase CRC risk, including notable examples such as *Fusobacterium nucleatum*, Enterotoxigenic *Bacteroides fragilis*, and pks^+^ *E. coli*. In recent years this work has expanded in scope to include aspects of host mutational status, intra-tumoral microbial heterogeneity, transient infection, and the cumulative influence of multiple carcinogenic bacteria after sequential or co-colonization. In this review, we will provide an updated overview of how host-bacteria interactions influence CRC development, how this knowledge may be utilized to diagnose or prevent CRC, and how the gut microbiome influences CRC treatment efficacy.

## Introduction

The human colon is colonized by a symbiotic community of microbes containing an estimated 10^14^ bacteria, similar to the total number of mammalian cells.^1^ Under healthy conditions, these commensal bacteria promote intestinal homeostasis by facilitating digestion, metabolic outputs, immune tolerance, and epithelial maturation or function. The advent of next-generation sequencing has allowed researchers to catalog how microbial community composition and function change during the progression of various gastrointestinal diseases, including colorectal cancer (CRC). Broadly, these studies have shown that there are specific bacterial taxa that are associated with protection against or promotion of CRC.^2–4^ For example, some bacteria potentially introduce cancer-initiating mutations by producing microbial genotoxins^5^ while others produce metabolites that interfere with core metabolic processes in cancer cells.^6^ Moreover, the bacterial species present within the intestinal tract alter local and systemic cellular or metabolic profiles to influence treatment efficacy.^7^ The wide impact of microbiota on tumorigenesis has resulted in its incorporation in the hallmarks of cancer.^8^ Such beneficial or deleterious host-microbe interactions are influenced by a complex metabolic environment, other microbial community members, host mutational status, immune landscapes, and single-cell heterogeneity. In this review, we will summarize the current evidence for several proposed carcinogenic bacteria in CRC, with specific consideration for how these cancer-promoting strains operate in various tumor developmental and mutational contexts. Then we will consider how polymorphic communities or species may find application in the clinic as tools for CRC diagnosis, prevention, and treatment modulation.

## Colorectal cancer etiology

An overwhelming majority of CRC cases (~80-85%) are not driven by hereditary mutation, a phenomenon termed sporadic CRC, suggesting that environmental determinants such as lifestyle, diet and microbial community trigger tumorigenesis. Sporadic CRC most commonly occurs after the acquisition of mutations in the *adenomatous polyposis coli* (APC) gene triggers the cascade of events leading to CRC.^9^ APC acts as a negative regulator of WNT/β-Catenin, a proliferative signaling pathway whose upregulation is associated with cancer development. Subsequent mutations in other tumor suppressor genes, such as tumor protein 53 (TP53) and Kirsten rat sarcoma viral oncogene homolog (KRAS), further promote malignant transformation.^10^ In approximately 15% of CRC patients, a heritable mutation or epigenetic silencing of mismatch repair genes (mismatch repair deficiency, MMRd) results in a disproportionate number of mutations in repetitive DNA satellites called microsatellite instability (MSI-high), that may increase the likelihood of CRC driver mutations.^11^ Alternatively, chronic inflammation can drive dysplastic transformation giving rise to colitis-associated cancer (CAC) that has been recently reviewed elsewhere.^12^ While the same driver mutations are implicated in CAC, the frequency and timing of these mutations is different, with mutations in TP53 typically preceding APC.^12^ Regardless of etiology, most CRC cells are characterized by the activation of pro-survival (e.g. nuclear factor-κB; NF-κB), proliferative (e.g. WNT/β-Catenin), or immunogenic (e.g. signal transducer and activator of transcription 3; STAT3) pathways.^13–15^ Most studies aimed at determining how bacteria may promote CRC utilize susceptible animal models generated to recapitulate these changes. These can include mice with germline mutations in *Apc* (*Apc^Min/+^*) or the addition of exogenous mutagens (azoxymethane; AOM) coupled with additional mutations in immunosuppressive genes (e.g. interleukin-10; *Il-10*) or the addition of inflammatory chemicals (dextran-sodium sulfate; DSS) to mimic CAC. While this discussion provides an overview of the models commonly discussed in this review, this description is by no means exhaustive. For a more detailed overview, readers should refer to prior reviews.^16,17^

## Proposed carcinogenic bacteria

**Figure 1.**
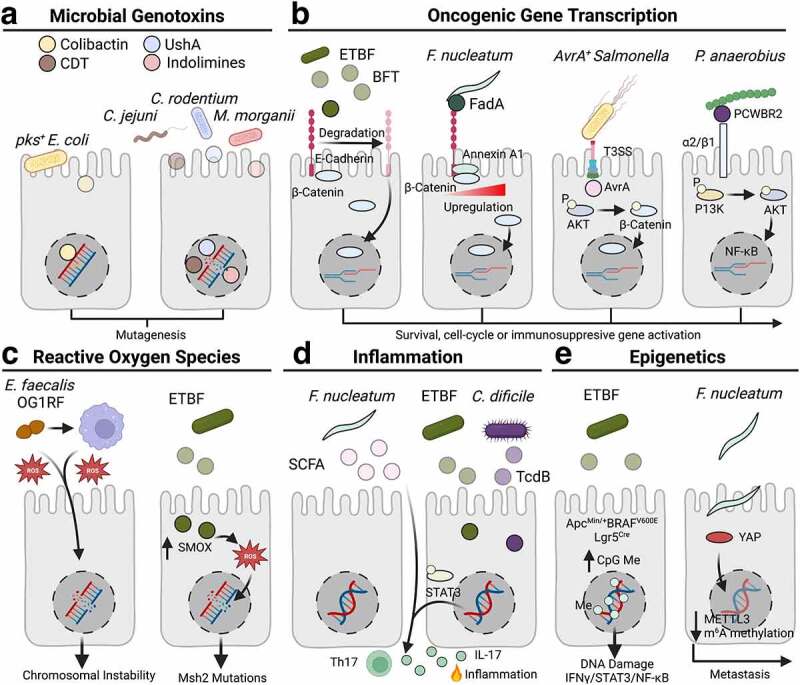
Carcinogenic mechanisms of intestinal bacteria. a) *pks^+^ Escherichia coli* produce a genotoxin known as colibactin, that induces interstrand crosslinks in host cells resulting in a defined mutational signature detected in colorectal cancer (CRC) genomes. The right panel shows several microbial genotoxins with nonspecific DNA degrading activity, including the cytolethal distending toxin (CDT) found in the human enteric pathogen *Campylobacter jejuni*, UshA in the murine bacteria *Citrobacter rodentium*, and indolimines isolated from a commenstal strain of *Morganella morganii* obtained from patients with inflammatory bowel disease. b) Enterotoxigenic *bacteroides fragilis* (ETBF) secretes a toxin known as the bacteroides fragilis toxin (BFT), that degrades E-cadherin promoting nuclear translocation of β-Catenin and the activation of proliferative signaling pathways to promote tumor formation. *Fusobacterium nucleatum* produces a membrane-bound Fusobacterium adhesin A (FadA) protein that binds to E-cadherin to upregulate expression of the Annexin A1/β-Catenin complex to activate proliferative signaling pathways. AvrA is a virulence factor produced by *Salmonella spp*. promoting epithelial adherence and persistent colonization in the gastrointestinal tract, while simultaneously activating AKT serine/threonine kinase 1 (AKT)-mediated β-Catenin phosphorylation, facilitating nuclear translocation and the activation of signaling pathways to promote proliferation and cell survival. *Peptostreptococcus anaerobius* are selectively enriched in CRC tissue, facilitated in part by the binding of an outer membrane protein putative cell wall binding repeat 2 (PCWBR2) to α2/β1 integrins overexpressed in cancer cells. This interaction promotes phosphoinositide 3-kinase (P13K) and AKT phosphorylation to promote cell proliferation. c) A superoxide producing strain (OG1RF) of the human pathogen *Enterococcus faecalis* causes chromosomal instability after infection in cell lines and intestinal ligation models, resulting from the production of reactive oxygen species (ROS). Alternatively, OG1RF infected macrophages elicit similar effects in human cell lines. On the right, infection with ETBF upregulates expression of a spermine oxidase that generates ROS and DNA damage in colonic epithelial cells. d) *F. nucleatum* produces short-chain fatty acids (SCFA) that bind to Free Fatty Acid Receptor 2 (Ffar2) receptors on T helper 17 (Th17) macrophages or an undetermined intermediate dendritic cell to stimulate interleukin 17 (IL-17) production. The human pathogens Enterotoxigenic *Bacteroides fragilis* (ETBF) and *Clostridioides difficile* (*C. difficile*) secrete toxins (the *Bacteroides fragilis* toxin, BFT, and *Clostridioides difficile* toxin B, TcdB, respectively) that promote signal transducer and activator of transcription 3 (STAT3) phosphorylation and the recruitment of IL-17 producing Th17 cells. In both cases, these microbes promote IL-17 mediated inflammation that contributes to neoplastic transformation. e) ETBF infection promotes hypermethylation in Apc^Min/+^BRAF^V600E^Lgr5^Cre^ mice, resulting in the formation of proximal tumors and activation of IFNγ gene signatures. *F. nucleatum* infection downregulates methyltransferase 3 (METTL3) expression in a Yes1 associated transcriptional regulator (YAP)-dependent manner to inhibit m^6^A RNA methylation, altering mRNA translation to promote cancer metastasis.

It is now well established that intestinal bacteria influence the intestinal homeostasis, which has led to the theory that these microbes may act as an environmental trigger for CRC. Bacteria may promote CRC through diverse mechanisms, and we have likely only characterized a small portion of bacteria that might modulate cancer risk. The identification of novel carcinogenic bacteria may come from a better understanding of the consequences of pathogenic infection, or perhaps more intriguingly, from the discovery of novel cancer-promoting activity of commensal microbes. For example, Cao et al.^18^ recently identified 18 commensal strains from patients with inflammatory bowel disease that produce DNA damaging small molecules. One strain investigated in depth, *Morganella morganii*, produces a novel genotoxic indolimine through a previously uncharacterized biosynthetic pathway, and promoted tumor formation in a chemically induced model of colitis associated cancer.^18^ Another layer of complexity arises when considering the cumulative bacterial exposure of an individual over a lifetime, during which microbial community composition and activity may fluctuate. When considering this scenario, an individual’s risk of developing CRC may be influenced by a series of successive microbial “hits”. Such a concept is supported by differential bacterial colonization and metabolic outputs across CRC staging.^19^ While each exposure may not cause cancer by itself, the cumulative effect of multiple encounters with cancer-promoting bacteria may outweigh the sum of its parts. The processes by which these microbes are thought to promote CRC risk are highly diverse, encompassing changes in genomic integrity, oncogenic signaling, cellular migration, inflammatory states, and epigenetic changes, among others (Figure 1). In this section, we will review these mechanisms in detail, highlighting key proposed carcinogenic species that may promote CRC development.

### Pks^+^ Escherichia coli

Certain strains of *Escherichia coli* (*E. coli*) carry a 54-kb biosynthetic gene cluster (BGC) found primarily in the B2-phylogroup called polyketide synthase (“*pks*” or “*clb*”) that encodes for a secondary metabolite named colibactin that causes DNA damage in mammalian cells.^20,21^ While *E. coli* commonly exists as a commensal constituent of the human microbiome,^22^ epidemiological evidence suggests that *pks^+^ E. coli* are more prevalent in stool or tissue samples CRC patients than those with inflammatory bowel disease (IBD) or healthy controls.^23–27^ Administration of *pks^+^ E. coli* in pre-clinical models of colitis-associated cancer^25,28,29^ promotes tumor formation with a concomitant increase in a phosphorylated histone variant associated with DNA damage (γH2AX) attenuated by the deletion of *pks* genes, suggesting that these bacteria promote tumor development mediated by the genotoxic effects of colibactin. Because somatic mutations may result from DNA damage, genotoxic agents are commonly implicated as sources of oncogenic transformation, and a case can be made for *pks^+^ E. coli* in this process. Chronic exposure of human organoids to *pks^+^ E. coli* generates a unique transcriptional signature characterized by single-base substitutions (SBS) or insertion-deletions (ID) within AT-rich DNA motifs, and similar signatures are identified in approximately 5% of metastatic tumors of predominately CRC-derived primary sites.^5^ This mutagenic activity is consistent with colibactin’s proposed structure characterized by two cyclopropane warheads that form interstrand DNA crosslinks (ICLs) by adenosine addition,^30,31^ linked by an unstable α-aminoketone susceptible to nucleophilic cleavage.^32,33^ Under mild conditions, an estimated >98% of the molecule is lost to 1,2-diketone degradation during isolation.^34^ This rapid degradation may explain why the genotoxic effects of *pks^+^ E. coli* require cell-to-cell contact^21^ and can be exacerbated by the addition of a mucolytic agent to cell culture^35^ or co-inoculation of *Apc^Min/+^* mice with biofilm-initiating enterotoxigenic *Bacteroides fragilis* (ETBF) that facilitate deeper mucosal invasion.^23,36^

While spatial *pks^+^ E. coli* distribution in the gut may influence the carcinogenic effects of colibactin, environmental regulation of *pks* genes and thus colibactin biosynthesis likely also plays a role.^37^ Genetic studies indicate that all genes involved in colibactin production are required for *pks*-associated genotoxicity,^21^ and multiple studies have shown that *pks* gene expression is influenced by inflammation,^28,29,38^ iron availability,^39–41^ microbial metabolites,^42^ oxygen availability^43^ and dietary oligosaccharides.^44^ These findings suggest that modifiable risk factors, such as diet, may influence the likelihood of individuals colonized by *pks^+^ E. coli* developing CRC. This hypothesis is supported by a recent study quantifying cancer risk associated with a western diet in patients with positive *pks* gene detection in formaldehyde-fixed paraffin-embedded samples gathered from 1175 patient records with dietary history.^45^ While a Western diet (i.e. high red meat and low dietary fiber consumption) only weakly correlated with CRC in a large cohort of male and female health professionals (Health Professionals Follow-up Study/Nurses Health Study, n = 134,775), the Western diet hazard ratio was significantly increased in patients with high microbial *clbB* expression.^45^ Associations such as this may explain why not all individuals colonized by *pks^+^ E. coli* develop CRC, despite the implication of colibactin as a carcinogenic metabolite.

Related to these findings, it remains unclear if colibactin’s role in CRC etiology is primarily related to tumor initiation, progression, or both. Several lines of evidence suggest that *pks^+^ E. coli* promote tumor-initiating mutagenesis. First, the tumorigenic phenotype observed in pre-clinical models is primarily characterized by an increase in tumor number rather than an increase in tumor size.^25^ Additionally, in CRC cases harboring the *pks^+^ E. coli* mutational signature, the highest proportion of mutations matching the *pks* motif (AAWWTT) occur within the APC gene (5.3%),^5^ the most commonly mutated gene in CRC patients. Finally, the evidence suggests that these mutational signatures are acquired during childhood^46^ and that *pks^+^ E. coli* may be acquired as early as the first month of life via mother-to-infant transmission.^47,48^ On the other hand, the prevalence of *pks*-associated mutational signatures is much lower (~5%) than the observed number of CRC patients colonized by *pks^+^ E. coli* (~55-60%), suggesting these bacteria may promote tumor progression through a non-mutagenic mechanism. For example, *pks^+^ E. coli* infection promotes a pro-tumorigenic microenvironment by generating a senescence-associated secretory phenotype in bystander cells,^49,50^ and reduces the number of cytotoxic T cells within the invasive margins of CRC tumor biopsies.^51^ Finally, while some studies have shown an increased prevalence of *pks^+^ E. coli* in normal tissue and early adenomas, a recent study found that *pks* genes were enriched only in stage IV tumors in a French cohort.^52^ Collectively, these studies implicate *pks^+^ E. coli* in CRC, but more work is required to fully understand the mechanistic role of colibactin itself in the multifaceted aspects of tumor initiation and cancer progression.

### Enterotoxigenic bacteroides fragilis

Enterotoxigenic *Bacteroides fragilis* (ETBF) strains produce a ~ 20 kDa toxin termed the *B. fragilis* toxin (*bft*), a zinc metalloprotease associated with intestinal disease and persistent colitis in humans.^53–55^ Moreover, several epidemiological studies have demonstrated a higher incidence of ETBF infection in CRC patients,^23,52,56,57^ suggesting *bft* may play a causative role in CRC. Pre-clinical studies utilizing the *Apc^Min/+^* mouse model have demonstrated that colonization with ETBF promotes colitis and the formation of distal colon tumors.^55,58,59^ In this model, tumor formation is inextricably linked to the immune system, dependent on the recruitment of T helper 17 (Th17) and CXC motif chemokine receptor 2^+^ (CXCR2^+^) myeloid derived suppressor cells driven by the activation of epithelial NF-κB/STAT3/interleukin 17 (IL-17) signaling cascades.^55,58,59^ In this context ETBF-associated tumorigenesis can be abrogated by IL-17 blockade or gene knockout,^55,58^ regulatory T cell (Treg) depletion,^59^ or epithelial STAT3 deletion.^55^ Additionally, *bft* intoxication directly activates transcriptional pathways associated with proliferation and stemness typical of multiple cancers. After binding to intestinal epithelial cells, *bft* facilitates E-cadherin degradation to activate WNT/β-Catenin signaling pathways to directly increase cell proliferation.^60^ Collectively, this data suggests that ETBF-mediated tumors are the result of multi-faceted pro-neoplastic changes to the epithelial microenvironment and cell-intrinsic processes.

DNA hypermethylation a hallmark of CpG methylator phenotype (CIMP) CRC cases.^61^ Recent evidence suggests that ETBF infection can alter epigenetic profiles in CRC cells, and that these changes may promote cancer cell growth. In murine organoids, ETBF infection upregulates expression of the JmjC-domain containing histone demethylase 2B (JMJD2B) that decreases Histone H3 Lysine 9 (H3K9me3) methylation levels in the Nanog homeobox promoter, a core regulator of embryonic stem cell proliferation.^62^ Consequently, these changes promote the preservation of a stem-like phenotype in organoids and promote tumor growth in HCT 116 xenografts.^62^ Although *btf* does not have direct genotoxic activity, several lines of evidence suggest that the epigenetic changes induced by ETBF infection may also promote the accumulation of DNA damage. In HT29 cells, 24-hour treatment with purified *bft* generates specific “*bft*-open” chromatin regions with enhanced gene expression.^63^ In a mouse model of CIMP (*Apc^Min/+^BRAF^V600E^Lgr5^Cre^*; BLM) ETBF colonization promoted tumor formation in the mid-proximal colon mimicking proximal tumors observed in CRC patients with *BRAF^V600E^* mutation, in contrast to the distal tumors canonically observed in *Apc^Min/+^*-ETBF mice.^64^ Moreover, ETBF colonization enhanced hypermethylation in BLM-ETBF tumors relative to distal *Apc^Min/+^*-ETBF tumors, with concomitant increase in interferon gamma (IFNγ)/STAT3/NF-κB signaling pathways.^64^ Notably, both BLM and *Apc^Min/+^* mice develop few spontaneous tumors, suggesting that ETBF can act as a microenvironmental trigger for CRC in multiple mutational contexts. Interestingly, in these cases hypermethylation was associated with a higher amount of DNA damage; a slightly higher single nucleotide variant (SNV) rate in *bft*-open chromatin,^63^ or a significantly higher number of γH2AX foci in BLM-ETBF tissue relative to *Apc^Min/+^*-ETBF tissue.^64^ These findings suggest ETBF infection may alter chromatin accessibility and indirectly facilitate DNA damage in CIMP CRCs. The genotoxic mechanism for such observations remains unclear, but they may be attributed to the generation of reactive oxygen species (ROS) mediated by *bft*. Purified *bft* upregulates the polyamine catabolic enzyme spermine oxidase to increase ROS production and subsequent γH2AX phosphorylation in HT29 cells, while administration of a polyamine catabolism inhibitor reduces ETBF-induced tumorigenesis in the *Apc^Min/+^* mouse model.^65^ Similarly, ETBF-induced tumors from a mismatch repair deficient mouse combining mutations in *Apc* and MutS homolog 2 (*Apc^Min/+^Msh2^fl/fl^VillinCre*) exhibit a higher mutational burden but no distinct signature, suggesting that ETBF colonization increases mutations attributed to deficient DNA-repair randomly throughout the genome, and consequently may increase the likelihood of a second truncating *Apc* mutation.^66^ These findings collectively reinforce the idea that ETBF colonization may generate epigenetic or genomic changes that, in specific contexts, may influence CRC etiology.

Given the high prevalence of ETBF in CRC cases, it is reasonable to consider their application as a CRC biomarker. The efficacy of such an approach is influenced by multiple factors, including the sampling method, reliability of detection, and the stage at which these bacteria can be detected. In most studies using tissue biopsies and assessing ETBF prevalence by quantitative real-time PCR (qPCR), ETBF abundance is positively correlated with tumor stage^56,57,62,67^ or equally enriched across tumor stage.^68^ However, in a recent study directly comparing the prognostic value of ETBF to four other CRC-associated bacteria in a French cohort, ETBF was the only species differentially enriched in fresh fecal samples from patients with early adenomas assessed by qPCR.^52^ These findings suggest ETBF detection in fresh stool may facilitate early detection of individuals with an increased risk of developing invasive disease or identify a subset of patients with early adenomas more efficiently than invasive colonoscopy-based methods.

### Fusobacterium nucleatum

*Fusobacterium nucleatum* (*F. nucleatum*) is commonly identified as a potential microbial carcinogen, a designation attributed to its frequent enrichment in CRC tumor tissue^69–71^ and a plethora of studies demonstrating that *F. nucleatum* colonization promotes tumor growth in *Apc^Min/+^* mice.^19,72–74^ The mechanisms underlying *F. nucleatum*-associated tumorigenesis have been reviewed thoroughly elsewhere,^71^ and include: the production of virulence factors such as Fusobacterium adhesin A (FadA) and fibroblast activation protein 2 (Fap2) that facilitate adhesion or colonization,^73,75^ activation of β-Catenin signaling pathways in cancer cells that promote tumor proliferation,^76,77^ the generation of immunosuppressive microenvironments that restrict anti-tumor immunity,^72,78^ and the promotion of colitis-associated cancer via an IL-17 or myeloid dependent mechanism.^73,74^ In addition to promoting the proliferation of primary cancer cells, several studies suggest colonization of CRC tissue by *F. nucleatum* also promotes metastasis.^79–83^ Enhanced metastatic capabilities may be attributed to exosomes secreted by infected CRC cells containing microRNAs (miRNAs) or cytokines that simultaneously reduce macrophage tumor infiltration and enhanced proliferative signaling cascades, and the production of these molecules correlates with *F. nucleatum* abundance in CRC patients.^79,80^ Furthermore, *F. nucleatum* infection may promote migratory phenotypes by altering epigenetic profiles via non-coding RNA^81^ or methyltransferase activity.^83^ However, an open and important question is whether *F. nucleatum* acts as an initiator of genomic or environmental remodeling to promote carcinogenesis,^84^ or if these bacteria are selectively enriched in neoplastic lesions and subsequently enhance malignant processes while supplanting tumor-initiating species.^85^ Epidemiological evidence suggests *F. nucleatum* are preferentially enriched in late-stage CRC tissue, an observation that may support their role as a tumor-potentiating bacterium, after initiation by another hereditary or environmental event. For example, Kostic et al.^72^ show that relative *F. nucleatum* abundance is significantly higher in stool samples from CRC patients with carcinoma relative to adenoma as assessed by qPCR analysis. Multiple studies have found a similar significant positive correlation between *F. nucleatum* abundance and CRC stage.^19,80^ Consistent with this hypothesis, *F. nucleatum* does not stably colonize the intestinal tract after oral administration in SPF mice, requiring daily gavage at a relatively high level (10^8^–10^9^ colony forming units, CFU^72,86,87^), but readily colonizes tumor tissue in an orthotopic CRC model after intravenous injection.^75,88^ This model of colonization is consistent with the theory that CRC-colonizing *F. nucleatum* strains originate in the oral cavity and translocate to intestinal tumor tissue after damage to the oral-intestinal barrier.^71,89^ Moreover, *F. nucleatum*-positive patient-derived xenografts (PDX) maintained *F. nucleatum* positivity for 29 weeks and eight sequential murine passages,^90^ highlighting the persistence of this bacteria in CRC tumors. In this study, *F. nucleatum* maintained a PDX growth-enhancing effect demonstrated by a reduction in tumor growth after metronidazole treatment.^90^ In contrast, colonization of germ-free mice with a mixture of six *fadA^+^, fap2^−^* or ^+^ *F. nucleatum* CRC clinical isolates did not promote tumor formation in *Apc^Min/+^* mice despite persistent *F. nucleatum* colonization quantified by fecal CFU.^91^ In a similar study no tumorigenic effect was observed after colonization of germ-free *Apc^Min/+^* mice with five unique CRC-derived *F. nucleatum* isolates after weekly gavage, despite successful colonization by four of the five strains.^92^ Collectively, this evidence supports a paradigm in which malignant transformation of colonic epithelial cells facilitates higher levels of *F. nucleatum* colonization.

Beyond these tumor-promoting effects, *F. nucleatum* colonization may influence clinical decision-making via its association with specific CRC subtypes or prognoses. Multiple studies have demonstrated that *F. nucleatum* abundance is enriched in premalignant lesions from MSI-high patients, CIMP patients, and patients presenting with sessile serrated adenomas.^93–96^ Moreover, *F. nucleatum* abundance is predictive of shorter survival in CRC patients.^96^ In such cases, simple clinical interventions aimed at reducing *F. nucleatum* abundance or inhibiting the production of virulence factors may be warranted. One practical approach may be the addition of an aspirin regimen, as aspirin (1–2.5 mM) inhibits *F. nucleatum* FadA/Fap2 expression, and the addition of a physiologically relevant dose of aspirin (200 ppm) to mouse chow inhibited *F. nucleatum*-induced tumorigenesis after daily gavage in *Apc^Min/+^* mice.^97^ These findings suggest that *F. nucleatum* may be a consistent biomarker for late-stage disease or premalignant lesions in a subset of CRC patients, and that therapeutic interventions aimed at reducing *F. nucleatum* abundance may improve patient outcomes.

### Enteric pathogens

The advent of large-scale metagenomic studies has ushered in a new age of microbiome research and resulted in the identification of candidate oncogenic bacteria associated with CRC. These approaches necessitate the species be present at the time of (or shortly preceding) diagnosis, and thus may inadvertently miss biologically relevant species that transiently promote cancer initiation at an earlier time point. Furthermore, such techniques may not detect significant changes in low-abundance microbiota. As a result, many bacterial species implicated as a risk factor for CRC are commensal species that persistently colonize the intestinal tract, while the effects of recurrent or transient infection with intestinal pathogens on CRC risk remains unclear. Pathogen infection may cause intestinal inflammation. Given that inflammation is a well-established CRC risk factor it is possible that such infections increase an individual’s risk for developing CRC. A summary of proposed CRC-associated enteric pathogens and their proposed carcinogenic mechanisms is given in Table 1.

**Table 1.** Enteric pathogens implicated in CRC.

Bacterium	Virulence Factors	Experimental/Epidemiological Evidence	Proposed Mechanism	Reference
*Salmonella spp.*	AvrA FliC	Increased incidence ratio and FliC-reactive antibodies in Dutch cohorts. Increased CRC risk after non-Enteritis or Typhimurium infections.	AvrA mediated colitis, activation of WNT/β-Catenin and STAT3 signaling pathways.	98,99,100,101,102,103
Pathogenic *Escherichia coli* *spp.*	Cif Cnf Cdt	EPEC detected in 55.9% of CRC patient biopsies, compared to 20.6% in healthy patient biopsies. *Cif* (7.9%), Cnf (36.7%), Cdt (8.2%) of CRC biopsies.	Activation of inflammatory signaling cascades, Cancer cell detachment and survival	26, 27, 104,105,106,107,108,109,110,111,112,113
*Clostridium difficile*	TcdB	Experimental validation of a carcinogenic strain from biofilm-positive fecal slurries	Increased WNT/β-Catenin signaling, pro-carcinogenic IL-17 mediated immune response	114
*Klebsiella pneumoniae*	Clb?	Experimental validation of tumorigenesis after infection in colitis-associated cancer models; mechanism unclear	Carcinogenic activity modulated by microbiome status	117
*Citrobacter rodentium*	UshA	Experimental validation, identification of UshA-induced mutational signature in CRC cases	DNA damage and mutagenesis after transient infection	118
*Campylobacter jejuni*	Cdt	Experimental validation of tumorigenesis after infection with a CRC clinical isolate	DNA damage	121

Abbreviations: colorectal cancer, (CRC); avirulence A, AvrA; flagellar structural protein, FliC; cycle inhibiting factor, Cif; cytotoxic necrotizing factor 1, Cnf1; cytolethal distending toxin, Cdt; Clostridium difficile Toxin B, TcdB; colibactin, Clb; UDP-sugar hydrolase, UshA; signal transducer and activator of transcription 3, STAT3; interleukin 17, IL-17.

The most thorough epidemiological evidence in this context comes from several studies assessing CRC occurrence in large cohorts of patients in the Netherlands after *Salmonella* infection. In one example, Mughini-Gras et al.^98^ cross-referenced population-based registries to investigate a cohort of 14,264 *Salmonella* cases for CRC incidence, finding an increased standardized incidence ratio (1.54) in early-onset CRC occurring primarily in the ascending/transverse colon. Furthermore, an updated analysis identified an increased risk of CRC more than 1-year post-infection with non-Enteritis or Typhimurium *Salmonella* serovars,^99^ and the detection of a *Salmonella* flagellar structural protein (FliC) antibodies is higher in Dutch and American CRC patients relative to healthy individuals.^100^ *Salmonella* colonization may also lead to chronic infection and inflammation that can exacerbate disease in this context. For example, the virulence factor avirulence A (AvrA) is necessary for chronic *Salmonella* colonization and resultant colitis.^101^ In a standard model utilizing the carcinogen AOM and DSS (AOM/DSS) to induce colitis-associated cancer, colonization with *avrA^+^ Salmonella* Typhimurium for 25–45 weeks simultaneously activates WNT/β-Catenin and STAT3 to promote tumor development.^102,103^ Similarly, multiple epidemiological studies have shown an increase in mucosal-invasive *E. coli* in patients with inflammatory bowel disease and colon cancer.^104–106^ Pathogenic *E. coli* strains can produce a variety of toxins (e.g. cycle inhibiting factor, Cif; cytotoxic necrotizing factor 1, Cnf1) that modulate the cell-cycle and are often found in CRC patients.^26,27^ However, unlike *pks*, the direct tumorigenic potential of these toxins has not been demonstrated in pre-clinical models. In contrast to the direct mutagenic activity attributed to *pks^+^ E. coli*, infection with pathogenic *E. coli* strains may contribute to tumor progression indirectly by promoting inflammation,^107^ senescence-associated secretory phenotypes,^108–111^ or cancer cell detachment and survival.^112,113^ Other enteric pathogens have been shown to promote CRC through similar inflammatory mechanisms. For example, a recent study by Drewes et al.^114^ isolated a strain of *Clostridioides difficile* (*C. difficile)* from biofilm-bearing CRC tissue that induced colonic tumorigenesis in germ-free and SPF *Apc^Min/+^* mice in a toxin-B dependent manner. *C. difficile* induced tumors occurred with concomitant activation of myeloid and IL-17 producing lymphoid cells, reminiscent of those observed after ETBF infection in the same model.^55,58,59^ Importantly, tumorigenesis was dependent upon consistent colonization for 10 weeks, as vancomycin administration via intraperitoneal injection beginning 1-week post-infection successfully inhibited microadenoma formation.^114^

The clinical relevance of these findings and epidemiological associations are difficult to parse because enteric pathogens are not typically identified in metagenomic studies from CRC patients, and symptomatic infections are ideally temporary after patients receive treatment. Thus, another clinically relevant question is whether transient infections by enteric pathogens might increase the risk of cancer development later in life. Temporally distinct associations such as these can be investigated by the detection of antibodies against specific pathogens in CRC patients^100^ but are limited by antigen specificity. Another approach can be to use tumor mutational signatures, in which exposure to a specific bacterium leaves a permanent genetic fingerprint in transformed host cells, most often as the result of exposure to microbial genotoxins. Such an approach has been described earlier using *pks^+^ E. coli*, but the *pks* gene island is distributed among other Enterobacteriaceae as well. For example, the opportunistic pathogen *Klebsiella pneumoniae* (*K. pneumoniae*) can also carry *pks* genes as part of a mobile genetic element also encoding yersiniabactin, and these genes are observed more frequently in hypervirulent strains.^115,116^ Moreover, these bacteria frequently colonize the gut during early life when the colibactin-associated mutational signature is thought to be acquired in colonic epithelial cells.^46^ Accordingly, a neonatal *K. pneumoniae* isolate promoted tumor formation in *Apc^Min/+^;IL-10^−/−^* mice, although this phenotype was still observed in pks*-deficient K. pneumoniae*.^117^ Thus, it is unclear whether the mechanism underlying *K. pneumoniae*-driven tumorigenesis is related to colibactin production in this model, but it is logical to theorize that other colibactin-producing microbes may elicit similar mutational signatures as *pks^+^ E. coli*. Liu et al^118^ recently demonstrated that transient infection with an attaching/effacing pathogen (*Citrobacter rodentium, C. rodentium*) producing a novel genotoxin previously characterized as a UDP-sugar hydrolase (UshA), generates mutational signatures that can be identified by whole-exome sequencing in resultant tumors. In this context, mice completely clear *ushA^+^ C. rodentium* infection 28-days post-colonization and *ushA^+^ C. rodentium* exposed mice develop a higher number of tumors approximately 4 weeks after clearance.^118^ These tumors have an increased proportion of single-nucleotide substitutions attributed to the COSMIC mutational signature SBS26 canonically attributed to mismatch-repair deficiency (MMRd), but not the related MMRd signature SBS15.^119,120^ Collectively, these findings suggest that exposure to microbial genotoxins may generate mutations that result in tumor formation long after bacterial clearance, and that the pattern of mutations in these cases may provide insight into transient carcinogenic events in the past. However, these applications may be limited, as in this case the observed mutations overlap previously defined signatures with etiologies proposed in high confidence (i.e. mismatch repair deficiency). In another case, some strains of the pathogenic bacterium *Campylobacter jejuni* (*C. jejuni*) produce a genotoxin known as the cytolethal distending toxin (*cdt*) that promotes colorectal cancer via its DNA-damaging activity.^121^ This toxin’s activity is mediated by its active subunit CdtB, which exhibits DNase-I-like activity,^122^ an enzyme that readily digests DNA with limited sequence specificity primarily dictated by chromatin accessibility.^123,124^ Whether CdtB exposure promotes a specific mutational signature that can be used to estimate its role in CRC initiation remains to be seen. Given the compound’s homology to DNase-I, it may be hypothesized that DNA damage after infection with CdtB-producing bacteria will result in an increase in overall mutational burden without a distinct identifiable signature. In cases like this, the methods of identifying a causative link between transient pathogenic infection and CRC risk remain difficult and require careful investigation.

### Enterococcus faecalis

*Enterococcus faecalis* (*E. faecalis*) is among the most prevalent commensal enterococci found in human stool and is enriched in fecal^125^ and formalin-fixed paraffin-embedded (FFPE)^67^ samples from CRC patients. However, whether *E. faecalis* colonization modules intestinal tumor development is debated^126^ and recent evidence suggests strain-specific differences may alternatively elicit pro- or anti-tumorigenic effects in normal intestinal epithelial and tumor cells.^127^

*E. faecalis’* carcinogenic activity is primarily attributed to its ability to directly damage host DNA via superoxide production, or through a macrophage-induced bystander effect. Infection with the superoxide-producing strain *E. Faecalis* OG1RF increased chromosomal instability in a hybrid hamster cell line harboring a human version of chromosome 11.^128^ Similar effects were observed after treating these cells with RAW264.7 macrophages that had been infected with OG1RF.^128^ These findings were later replicated in studies showing more anaphase bridges and aneuploidy in a CRC cell line HCT 116 after direct culture with *E. faecalis* or infected macrophages.^129^ Moreover, γH2AX foci were observed in colonic epithelial cells in a 6 h intestinal ligation model after direct mucosal exposure of *E. faecalis*.^129^ In both studies,^128,129^ these effects were abrogated by the addition of a superoxide dismutase, suggesting that resultant DNA damage and chromosomal instability resulted at least in-part from reactive-oxygen species. Interestingly, while testing various free radical scavenging molecules, Wang & Huycke^128^ noticed that an inactive control metabolite, γ-CEHC, also inhibited chromosomal instability in these cells without reducing superoxide concentrations in bacterial cultures. Instead, this molecule’s protective function was attributed to its inhibition of cyclooxygenase-2 (COX-2) signaling, a key inflammatory mediator in active macrophages upregulated during *E. faecalis*-associated colitis.^130^ Consistent with these studies, Wang et al.^131^ later showed that OG1RF-polarized macrophages induced heritable mutations in a normal colonic epithelial cell line (YAMC), and that allografts of these cells developed into poorly differentiated tumors in immunodeficient mice. These effects are observed with a concomitant increased expression of intestinal stem cell markers (e.g. doublecortin like kinase 1, Dclk1) and the activation of WNT/β-Catenin signaling pathways.^130–132^ In addition to activating proliferative signaling pathways, epidemiological evidence has linked *E. faecalis* colonization to distinct genomic and transcriptomic profiles in patients. These associations are primarily characterized by increased detoxification enzymes in pre-cancerous lesions (e.g. glutathione S-transferase alpha 4, GSTA4^133^) or increased inflammatory signaling pathways in *E. faecalis*-colonized CRC cases.^134^

Most of these studies utilize a single strain of superoxide producing *Enterococcus faecalis*, OG1RF, while strain diversity in the human gut microbiome may be very high. Several recent studies have focused on identifying strain-specific virulence factors that may elicit different phenotypes in colonic epithelial cells. For example, a collagenolytic rodent-derived *E. faecalis* strain (E2) promotes anastomotic tumor formation 21 days after surgical resection and delivery of bacteria via enema in mice fed a Western diet.^135^ These tumors can be attenuated by antibiotic depletion or inhibition of microbial collagenase activity by adding a phosphate carrier compound (Pi-PEG) to the drinking water.^135^ Consistent with these findings, Williamson et al.^136^ found that the human-derived strain *E. faecalis* V583 promotes invasion and migration in a human colon cancer cell line dependent on its collagenolytic activity of a secreted gelatinase (*gelE)*. This collagenolytic activity may also facilitate the translocation of immunogenic bacterial metabolites facilitating tumor progression at distant sites. Such a mechanism was recently described for V583 in a murine model of hepatocellular carcinoma.^137^ In this model, administration of V583 producing a related gelatinase (GelA) promoted liver tumor formation dependent upon myeloid differentiation primary response 88 (Myd88)-dependent innate immune signaling and increased *gelA*-dependent gut permeability.^137^ Moreover, liver dysfunction and unbalanced gut bile-acid concentrations may selectively promote *E. faecalis* expansion in the intestine.^137^ In contrast, some *E. faecalis* strains have proposed probiotic function primarily attributed to the bacterium’s lactic acid fermenting activity. At least one study found a decrease in culturable *E. faecalis* strains from CRC patients relative to healthy donors using a combination of culture-based matrix-assisted laser desorption/ionization-time of flight (MALDI-TOF) identification and culture-independent qPCR analysis.^127^ Pre-fermented media from three *E. faecalis* strains isolated from healthy donors inhibited the growth of human CRC cell lines *in vitro*, while no effect was observed in two CRC-derived strains.^127^ Moreover, these authors found a decrease in *E. faecalis* prevalence in a small cohort of CRC patients relative to healthy subjects using a combination of culturomics/MALDI-TOF identification and real-time PCR analysis.^127^ If lactic-acid production facilitates anti-tumorigenic activity in this species, it is reasonable to theorize that this probiotic function requires metabolically active *E. faecalis* colonization in the intestine. This hypothesis is consistent with a study demonstrating that a heat-killed probiotic *E. faecalis* strain (EC-12) did not significantly reduce colonic tumor development in an *Apc^Min/+^* mouse model, although a slight reduction in polyp β-Catenin staining was reported.^138^ Collectively, this evidence suggests that the pro- or anti-tumorigenic consequence of *E. faecalis* colonization in the gut may be dictated by strain-specificity, the presence/absence of specific virulence factors, and metabolic activities associated with live bacteria.

### *Parvimonas micra* & *Peptostreptococcus spp.*

*Parvimonas micra* (*P. micra*) and *Peptostreptococcus spp*. are gram-positive anaerobic cocci that commonly exist as commensal members of the intestinal or oral microbiome. Both genera are enriched in CRC patients particularly in late-stage tumors,^19,52,94,139^ and incorporation of *P. micra* and *Peptostreptococcus stomatis* (*P. stomatis*) into a four-species biomarker panel facilitated noninvasive CRC diagnosis with high accuracy.^140^ Furthermore, both genera occupy a similar ecological niche and associate with related molecular CRC subtypes. For example, *P. micra* and *P. stomatis* were enriched in CRC patients from consensus molecular subtype 1 (CMS1) cases characterized by CpG-island hypermethylation, microsatellite instability, and serrated adenoma presentation.^141^ MSI-high tumors have high intra-tumoral microbial diversity that increases with tumor stage, with *Parvimonas* and *Peptostreptococcus* being among the most highly variable colonizers.^94^ In this case and others^142,143^ these bacteria are frequently found in mucosal biopsies from CRC tumors relative to adjacent normal tissue or healthy controls. Collectively, these findings suggest that these strains may share similar niche-specific colonization of tumor tissue in the gut, and this hypothesis is borne out by several studies using CRC cell lines and *Apc^Min/+^* mouse models. For example, *P. micra* and *Peptostreptococcus anaerobius* (*P. anaerobius*) adhere to the CRC cell-line HT-29 after co-culture and promote proliferative signaling markers (e.g. β-Catenin, PCNA; proliferating cell nuclear antigen) although a precise mechanism for these observations is not clear.^139,143^ A *P. micra* strain isolated from a CRC patient increased tumor formation in *Apc^Min/+^* mice with a concomitant upregulation of proliferative signaling pathways, but this phenotype required strain delivery by oral gavage at 10^8^ CFU every 3 days for 10 weeks.^143^ Germ-free *Apc^Min/+^* mice administered a single dose of *P. micra* did not form tumors but did generate a higher level of the proliferative markers Ki67 and PCNA in epithelial nuclei.^143^ Similarly, *P. anaerobius* increased the number of colonic tumors in *Apc^Min/+^* mice after a daily gavage of 10^8^ CFU for 10 weeks^144^ or high-grade dysplasia after a single gavage at the same dose after antibiotic treatment in mice administered AOM.^139^ Preferential adherence to CRC cells may facilitate these phenotypes, as *P. anaerobius* express a surface membrane protein, putative cell wall binding repeat 2 (PCWBR2), that binds integrin α2/β1 overexpressed in CRC cells to active phosphoinositide 3-kinase-AKT serine/threonine kinase 1 (p13k-AKT) proliferative signaling pathways.^144^ Consistent with this hypothesis, intraperitoneal administration of a peptide that binds integrin α2/β1 attenuated *P. anaerobius*-induced tumor formation.^144^ As in animal models of *F. nucleatum*, the requirement for frequent gavage of these strains and the lack of tumorigenicity in germ-free models suggests that these microbes may preferentially colonize cancer tissue rather than initiate tumor formation. Epidemiological associations with CIMP/MSI-high phenotypes suggests that higher mutational burden may somehow facilitate host-microbe interactions in these genera.

## Intestinal bacteria in CRC diagnosis, prevention, and treatment

The goal of microbiome research in the context of CRC is to integrate this knowledge to improve clinical outcomes. Currently, most studies focus on leveraging bacterial communities for one of three applications: noninvasive diagnostic methods, the prevention of CRC by restoring microbial dysbiosis or providing probiotic supplementation, or the modulation of gut microbes to boost therapeutic responses. Although progress has been made in each of these areas, major barriers remain before such techniques are routinely utilized in the clinic. In this section, we will discuss these advances as well as some of the barriers that must be addressed to realize the potential of microbiome-based medicine.

### Multi-omics models for CRC diagnosis

**Figure 2.**
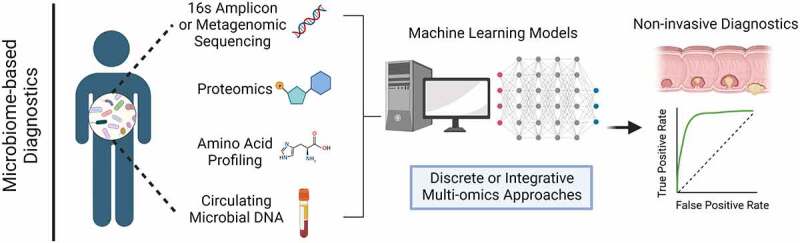
Multi-omics microbiome-based diagnostic methods. Microbiome-based diagnostic models typically utilize three primary technologies derived from patient fecal samples: 1) 16s amplicon or metagenomic sequencing to determine microbial taxa, 2) Liquid chromatography-mass spectrometry (LC-MS) metabolomics/proteomics to identify microbially derived metabolites, and 3) High-performance liquid chromatography (HP-LC) profiling of amino stool amino acid profiles. A fourth and more recently proposed approach amplifies circulating microbial DNA from patient blood or plasma samples, followed by stringent computational filtering. In all methods, marker selection is conducted using machine learning models to identify discriminating markers correlating with tumor stage, and accuracy can be improved by integrating one or more of these datasets.

CRC diagnosis primarily focuses on two strategies: the early identification of asymptomatic patients via routine colonoscopy, and the identification of symptomatic patients via clinical presentation.^145^ Given that CRC is often associated with the enrichment or depletion of specific bacterial genera or species, it is possible that a diagnostic set of microbes, microbially derived metabolites, or bacterial gene signatures may provide clinical information without the need for invasive procedures.^146^ Fecal metagenomics has shown a CRC-associated microbial signature primarily characterized by the increased relative abundance of several core species, namely: *F. nucleatum, P. stomatis, P. micra, Solobacterium moorei*, and *Bacteroides fragilis*.^147,148^ Recent work has focused on developing a set of universal CRC biomarker species that may be used in diverse cohorts with unique ethnicities or geographical origins, a difficult task given the non-uniformity of the microbiome in healthy individuals or cancer patients. Notably, Yu et al.^148^ present a panel of four microbial genes from these bacteria identified in Chinese patients with high diagnostic accuracy (73%) in validation cohorts from Denmark, France, and Australia. These results are consistent with a later study that utilized a machine-learning method to identify globally conserved bacterial CRC biomarkers from metagenomic sequencing data using three independent cohorts in the United States, France, and China.^149^ These studies suggest that fecal metagenomic screening may be a viable noninvasive screening method or could be used in conjunction with standard screening methods (such as a fecal occult blood test) to improve accuracy. However, these approaches have important limitations. For one, even the most effective models operate with a predictive capacity between approximately 73–85%. Related to this, the diagnostic accuracy of these models typically inversely correlates with the cancer stage,^147,148^ limiting the efficacy of using microbial biomarkers to identify patients before advanced disease. Finally, these approaches utilized fecal samples that are not routinely collected from patients. Poore et al.^150^ provide a proof-of-concept study that addresses some of these limitations by filtering microbial reads from metagenomic sequencing of CRC patient blood samples, providing more accessible material for microbial diagnostics. After *in silico* contaminant removal and training, machine-learning models were able to predict CRC cases with high accuracy using circulating microbial DNA from blood or plasma, in some cases discriminating between stage I/II cancers before circulating tumor DNA.^150^ Moreover, combining microbial biomarkers with genomic and metabolomic signatures may enhance screening or clinical decision-making. A recent study^151^ showed that patients with no disease, adenomas, or CRC could be differentiated with high accuracy from a set of discriminating microbial, amino acid, or proteomics features in patient feces. This multi-omics approach identified correlations between CRC-enriched microbes and biologically relevant metabolites, and outperformed traditional fecal immunochemical tests currently used in the clinic.^151^ Continued advancements in these techniques may facilitate the routine inclusion of microbial signatures in the clinic as part of a holistic diagnostic approach and allow for more accurate noninvasive cancer diagnosis or staging (Figure 2).

A major limitation of these approaches is that they all require the application of next-generation sequencing or proteomic analyses and complex machine-learning algorithms (Figure 2), techniques that require expensive dedicated equipment. Moreover, using these techniques in the clinic will require employing expert personnel, particularly with respect to data analysis and the application of predictive machine-learning models. Incorporating these methods in routine clinical diagnostics would require a large economic investment and is unlikely to become commonplace until significant technological advancements reduce the cost of these approaches.

### Probiotics and prebiotics

**Figure 3.**
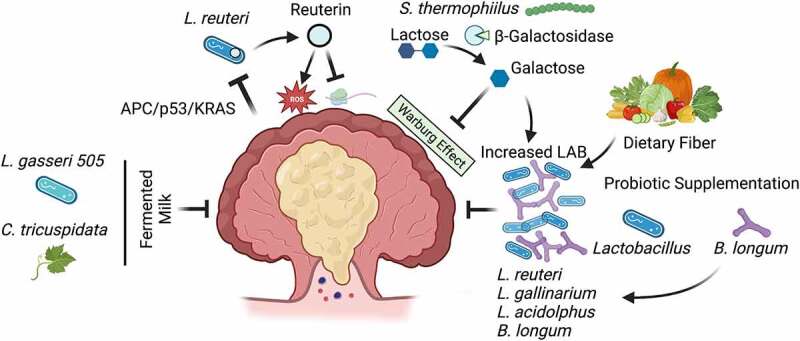
Lactic acid bacteria inhibit colorectal cancer tumorigenesis. Colorectal cancer (CRC) is characterized by a reduction in lactic acid bacteria (LAB). In a murine model of adenomatous polyposis coli/tumor protein 53/Kirsten rat sarcoma viral oncogene homolog (APC/p53/KRAS) mutant CRC, host metabolites directly inhibit *Lactobacillus reuteri* (*L. reuteri*) growth and the production of the microbial metabolite reuterin, that inhibits protein translation and generates cytotoxic reactive oxygen species in CRC cells to restrict cell growth. *Streptococcus thermophilus* (*S. thermophilus*) secretes the enzyme β-Galactosidase that produces galactose, that inhibits oxidative phosphorylation and Warburg metabolism in CRC cells. Various LAB inhibit tumor growth through a variety of indeterminate mechanisms, and the CRC-associated depletion of these species can be offset by increased abundance of galactose, dietary fibers, or the presence of other probiotic species. Finally, LAB may ferment dietary components such as *Cudrania triscuspidata* (*C. tricuspidata*) to produce antioxidants that restrict tumor growth.

The healthy gut microbiome is typically characterized by the presence of beneficial species that degrade complex polysaccharides derived from indigestible dietary fibers to produce lactic acid (lactic acid bacteria, LAB) and other metabolites that promote intestinal homeostasis.^152^ CRC-associated dysbiosis is characterized both by enrichment of CRC-associated bacteria and a depletion of probiotic LAB^153^ (e.g. *Bifidobacterium, Lactobacillus, Streptococcus*). While there are many associative and mechanistic studies implicating carcinogenic bacteria in CRC initiation or progression, there are fewer studies investigating the preventive potential of probiotic microbes. In the context of CRC tumor prevention, *Lactobacillus* is perhaps the most thoroughly studied genus. For example, daily administration of *Lactobacillus acidophilus* (*L. acidophilus*) probiotics to the drinking water of AOM-treated BALB/c mice inhibits tumor development.^154^ These findings are consistent with another study showing that oral gavage of *L. acidophilus* lysates every other day reduced tumor burden in an AOM/DSS BALB/c mouse model.^155^ These effects may be attributed to the production of exopolysaccharides and other metabolites identified in related *Lactobacillus spp*. that promote anti-tumor immunity or directly induce cancer cell apoptosis.^156,157^ Similar anti-tumorigenic effects have been observed after colonization by other LAB such as *Bifidobacterium longum* (*B. longum*)^158^ and *Streptococcus thermophilus* (*S. thermophilus*)^159^ (Figure 3).

While the specific factors resulting in LAB depletion in CRC patients are unclear, the current evidence suggests that the relative abundance of these bacteria is influenced by the tumor microenvironment, the presence of cross-feeding microbes, and the patient’s diet. Using a tamoxifen-inducible model with sequential mutations in key CRC driver genes (APC/p53/KRAS), Bell et al.^6^ show that homocysteine-degrading metabolites enriched in stool samples from triple mutant tumors inhibit *Lactobacillus reuteri* (*L. reuteri*) growth. Reuterin derived from these bacteria inhibited ribosomal biogenesis and induced cytotoxic ROS in multiple CRC cell lines, but not the normal colonic epithelial cell line NCM460 or HeLa cells.^6^ Moreover, reuterin supplementation in APC/p53/KRAS-mutant mice increased tumor-associated ROS and dysplastic transformation^6^ (Figure 3). The depletion of probiotic strains like *L. reuteri* in pre-clinical models of CRC can be offset by the presence of other probiotic strains. For example, oral administration of a high dose of *B. longum* (four 14-day cycles with 7 days between, 1.5 × 10^10^ CFU) inhibited tumor initiation in rats treated with AOM/DSS and consequently ameliorated *Lactobacillus* depletion observed in AOM/DSS rats not gavaged with *B. longum*.^158^ Similarly, daily gavage with *Lactobacillus gallinarium* (*L. gallinarium*) reduced tumor number and size in both *Apc^Min/+^* and AOM/DSS mouse models, with a concomitant increase in other probiotic species like *L. reuteri*.^160^ While these findings suggest that colonization with LAB promotes the proliferation or persistence of other probiotic species, the underlying mechanism remains unclear (Figure 3). LAB may promote the outgrowth of other probiotic strains simply by reducing the relative abundance of non-LAB species with an advantage in competition for limited resources. Alternatively, metabolic interactions between these microbes may favor the entire group. Another probiotic strain, *S. thermophilus*, suppresses CRC tumor growth in *Apc^Min/+^* mice by producing the enzyme β-Galactosidase that inhibits Warburg metabolism, a mode of aerobic glycolosis used for respiration in cancer cells.^159^ β-Galactosidase metabolizes a specific glucose moiety of lactose to produce galactose, and the addition of *S. thermophilus* or galactose to *Apc^Min/+^* mice increased the relative abundance of other probiotic species from the genera *Lactobacillus* and *Bifidobacterium*^159^ (Figure 3). These findings suggest that, in this context, *S. thermophilus* promotes the expansion of probiotic bacteria through a metabolic interaction. Furthermore, the *Apc^Min/+^* model used in this study developed relatively few colonic tumors (~1-2), suggesting competition within the tumor environment would not lead to pronounced changes in bacterial abundance.

Because nearly all probiotic strains discussed here ferment dietary fiber as a primary nutrient source, it is reasonable to consider dietary interventions aimed at enriching the abundance of these bacteria as a potential means of CRC prevention. One set of studies has used such an approach to demonstrate the efficacy of combining dietary modification with the anti-tumorigenic properties of *Lactobaillus gasseri* 505 (*L. gasseri* 505) in pre-clinical models. *L. gasseri* 505 was identified from a screen of neonatal isolates capable of fermenting milk supplemented with leaf extract from *Cudrania tricuspidata* (*C. tricuspidata*), a traditional herbal remedy in Asia, to produce lactic acid and a variety of antioxidant compounds.^161,162^ Accordingly, treating mice with *C. tricuspidata* leaf extract in milk fermented with *L. gasseri* 505 reduced tumor number and inflammatory gene expression in an AOM/DSS mouse model, while causing an expansion of *Bifidobacterium spp*.^163^ Several early-stage clinical trials have been conducted to assess how short-term dietary fiber interventions influence the gut microbiome in humans. In one study utilizing a cohort of undergraduate students, individuals were directed to increase fiber intake from ~21 g/day to 46.4 g/day for two weeks.^164^ Shannon diversity decreased after dietary intervention, but the relative abundance of *Bifidobacterium* increased, and *Bifidobacterium* abundance positively correlated with *Lactobacillus*.^164^ A second study utilized a random cross-over design in which the same individuals were given varying concentrations of inulin, arabinoxylan, or mixed fibers.^165^ In this study, a similar decrease in Shannon diversity correlating with the abundance of fiber added to the diet was observed, and the relative abundance of *Bifidobacterium* and biochemical pathways involved in fructose metabolism were observed after inulin administration.^165^ Collectively, these findings suggest that even short-term dietary interventions may promote the outgrowth of beneficial probiotic species in humans and that these bacteria exhibit anti-tumorigenic activity in pre-clinical models. Whether such an approach can reduce CRC risk in humans remains to be seen. Of note, and contrary to typical association between healthy status and high microbiota diversity, both studies reported a reduction in gut microbe biodiversity after short-term increased fiber intake, highlighting the unpredictable nature of dietary interventions in diverse human populations. A more precise clinical approach may be to directly administer bacterial metabolites responsible for these prophylactic effects, possibly from fermented supplements, as future CRC-preventive therapies.

### The influence of gut bacteria on CRC treatment

**Figure 4.**
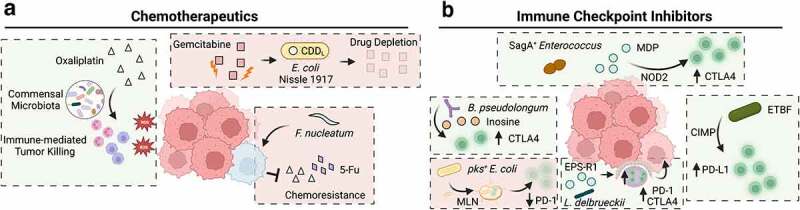
The impact of intestinal bacteria on colorectal cancer treatment. a) In mice harboring a commensal microbiota, neutrophils and macrophages invade tumors and produce reactive oxygen species (ROS), enhancing the tumor-killing effect of oxaliplatin. In mice administered antibiotics, the number of infiltrating immune cells and ROS-mediated cytotoxicity is reduced. In the presence of commensal bacteria (such as *Escherichia coli* Nissle 1917) harboring the long form of a cytidine deaminse (CDD_L_), gemcitabine levels are reduced after degradation by this bacterial enzyme, resulting in a reduced therapeutic response. Similarly, infection with *Fusobacterium nucleatum* confers resistance to oxaliplatin and 5-Fu by downregulating miRNAs that suppress autophagy and survival signaling. b) Several bacterial species have been linked to enhanced immunotherapy response in murine models of CRC or MC-38 xenografts. The species *Bifidobacterium longum* produces a metabolite inosine, which activates tumor-infiltrating T cells and exacerbates tumor killing after anti-CTLA4 treatment. *Enterococcus spp*. harboring the secreted antigen A (sagA) gene generate high levels of muramyl dipeptide (MDP) that activates nucleotide-binding oligomerization domain-containing protein 2 (NOD2) signaling pathways in colonic epithelial cells that drives immune recruitment and synergizes with anti-CTLA4 treatment. In a murine model of CpG island methylator phenotype (CIMP) CRC, infection with Enterotoxigenic *Bacteroides fragilis* promotes the recruitment of interferon gamma (IFNγ)-producing CD8^+^ T cells to enhance anti-PD-1 efficacy. *Lactobacillus delbrueckii* subsp. *bulgaricus* exopolysaccharides (EPS-R1) promote the activation of CCR6^+^CD8^+^ T cells in intestinal Peyer’s patches, as well as the number of IFNγ producing CD8^+^ tumor infiltrating cells, promoting the efficacy of anti-PD-1 and anti-CTLA4 treatment. Conversely, *pks^+^ E. coli* can migrate to mesenteric lymph nodes (MLN) and reduce systemic levels of CD3^+^ and CD8^+^ T cells, as well as the number of these cells observed in invasive tumor margins and reduces anti-PD-1 treatment efficacy.

Adjuvant chemotherapy (5-fluorouracil, 5-FU; oxaliplatin) is the recommended treatment option for advanced CRC cases that cannot be completely resolved by surgical excision.^166^ Medication use has a profound influence on gut bacterial community structure and metabolism,^167^ suggesting that the intestinal microbiome may modulate the efficacy of CRC therapies. One early study from Iida et al.^168^ demonstrated that antibiotic treatment significantly reduced the efficacy of oxaliplatin, a platinum-based chemotherapeutic, in a pre-clinical model using MC38 xenografts. Interestingly, antibiotic treatment had no effect on the proportion of platinum-bound DNA but rather reduced ROS generation by tumor-infiltrating CD11b^+^Gr-1^hi^ neutrophils and F4/80^+^Gr-1^int^ macrophage-like cells^168^ (Figure 4a). This finding suggests that commensal microbes in the normal gut biota regulate therapeutic efficacy by maintaining immune homeostasis. Alternatively, the outgrowth or persistence of deleterious species may inhibit these processes. Epidemiological evidence suggests that the persistence of *F. nucleatum* in patients undergoing neoadjuvant chemoradiotherapy is associated with a reduced rate of relapse-free survival (Hazard ratio = 7.5) over 140 months, with a concomitant reduction in tumor-infiltrating CD3+ and CD8 + T cells.^169^ Consistent with these findings, *F. nucleatum* is enriched in patients that exhibit a poor clinical response to 5-FU treatment, and *F. nucleatum* infection in HCT 116 and HT29 cell lines inhibits 5-FU mediated apoptosis via a toll-like receptor 4 (TLR4)/baculoviral IAP repeat containing 3 (BIRC3)-dependent mechanism in CRC xenograft models.^170,171^ Collectively, these data show that intestinal dysbiosis may influence platinum- or nucleoside-based chemotherapeutics (Figure 4a). The current evidence suggests that these effects are largely driven by disruption of immune homeostasis resultant from altered gut microbiota, although other mechanisms still need to be explored in more detail. For example, intravenous injection of *E. coli* Nissle 1917 confers gemcitabine resistance in MC26 xenografts dependent on expression of a long isoform of the enzyme cytidine deaminase (CDD_L_)^172^ (Figure 4a). In contrast, CDD_L_-deficient *E. coli* did not confer resistance to oxaliplatin.^172^ In this case, the reduced therapeutic efficacy was attributed to bacterial metabolism of the drug itself, as incubated CDD_L_^+^ strains with gemcitabine in minimal media led to nearly complete depletion of detectable drug within 4 hours.^172^ Whether 5-FU therapeutic efficacy is influenced by bacteria-mediated drug depletion in CRC patients remained to be established. Thus, the gut microbiome may influence chemotherapeutics by direct biotransformation of the active compound or by indirectly inhibiting the drug’s cytotoxic mode-of-action.

Immune checkpoint inhibitors (ICI) rely on the host immune system to mediate their anti-tumoral effect. Given that gut microbes influence local and systemic immune responses, many recent studies have investigated how intestinal bacteria modulate ICI therapies. For example, fecal microbiota transplant (FMT) from responders has been shown to sensitize ICI non-responsive tumor xenografts in pre-clinical models^173–176^ and human patients.^177,178^ Establishing a consistent mechanism for these observations has proven difficult, with responder-enriched taxa (most commonly: *Akkermansia spp., Bifidobacterium spp., Roseburia spp., or Faecalibacterium spp*.) varying across cohorts and cancer type.^179–181^ How microbial communities influence ICI response in CRC patients is less clear. CRC patients typically exhibit a poor response to ICI, with the exception being MSI-high tumors with higher mutational burdens, and thus a higher variable antigen presentation more likely to engage anti-tumor immune responses.^182^ MSI-high CRC can result from germline mutations in mismatch-repair associated genes or hypermethylation of the *MLH1* promoter; accordingly, MSI-high CRC is often associated with CIMP subtypes. Thus, an interesting question is whether gut microbes boost ICI efficacy in this context. DeStefano Shields et al. show that in a model of CIMP colon cancer (BLM, previously discussed), ETBF colonization increases the number of tumor-infiltrating IFNγ-producing CD8^+^ T cells relative to *Apc^Min/+^*-ETBF mice, and that anti-programmed death-ligand 1 (anti-PD-L1) is effective in reducing tumor burden in these animals^64^ (Figure 4b). To determine if microbiota composition can boost ICI efficacy in microsatellite stable (MSS) tumors, Mager et al.^183^ monitored treatment response and microbiome composition in an AOM/DSS model of colitis-associated cancer along with a heterotopic MC38 xenograft model. The authors found that *Bifidobacterium pseudolongum* (*B. pseudolongum*) mono-colonization enhanced intratumoral CD8^+^ T-cell activation while significantly enhancing the effects of ICI therapy^183^ (Figure 4b). While this bacterium only induced modest tumor growth inhibition in the absence of ICI, anti-cyototxic T-lymphocyte associated protein 4 (anti-CTLA4) treatment increased intratumoral *B. pseudolongum* abundance and systemic inosine levels, a *B. pseudolongum* metabolite that activated T_H_1 differentiation factor expression.^183^ Moreover, ICI treatment enriched the intratumoral abundance of these species in multiple mutational contexts, including: *Msh2^LoxP/LoxP^;Villin-Cre* and *Apc;^2lox14/+^Kras;^LSL-G12D/+^Fabpl-Cre* derived tumors.^183^ Several other studies have identified bacteria associated with ICI efficacy in murine xenograft models, most often utilizing the MSS cell line MC38. For example, Griffin et al.^184^ demonstrated that expression of a peptidoglycan hydrolase (secreted antigen A, SagA) in *Enterococcus spp*. facilitates the release of muramyl dipeptides that activate NOD2 signaling pathways and the expression of proinflammatory NF-κB genes. These changes promote tumor-infiltration by CD45^+^ and CD8^+^ cells, while enhancing the efficacy of anti-CTLA4 treatment in MC38 xenografts^184^ (Figure 4b). In some cases, the metabolite responsible for synergizing with immune checkpoint inhibitors can be isolated and administered as a bacteria-free adjuvant. For example, a recent study showed that exopolysaccharides derived from *L. delbrueckii* subsp. *bulgaricus* can activate CD8^+^ T cells in Peyer’s patches that preferentially infiltrate CCL20 positive tumors and promote the therapeutic efficacy of anti-CTLA4 or anti-programmed cell death protein 1 (anti-PD-1) treatment^185^(Figure 4b). In contrast, *pks^+^ E. coli* have been shown to translocate to the mesenteric lymph nodes where they lower the abundance of cytotoxic T cells, which in turn reduces immune cell invasion in tumor tissue margins and inhibits anti-PD-1 treatment in MC38 tumor-bearing mice^51^ (Figure 4b). This important area of research may begin to elucidate several important aspects of CRC treatment. Specifically, these studies suggest that a patient’s treatment response can be modulated by the presence or absence of specific bacterial species or microbial metabolites.

## Discussion

Systematic efforts to characterize the human microbiome began over a decade ago and were closely followed by an innumerable number of studies cataloging associations between changes in microbial community structure and human diseases such as CRC. These approaches have identified several bacterial species that are more frequently found in CRC patients, many of which have demonstrable carcinogenic effects when administered to susceptible murine models of CRC or colitis-associated cancer. These effects may be attributed to direct host-microbe interactions that activate proliferative^73,144^ or immunomodulatory^55,58,59^ signaling pathways. Alternatively, bacteria may produce toxins or metabolites that similarly activate oncogenic gene expression, induce epigenetic changes,^63,83^ or directly generate mutations in cancer-driver genes through their genotoxic activity.^5,186^ It is becoming clearer that the oncogenic potential of such microbes is influenced by the complex milieu of host- and microbe-derived factors occurring in the microenvironment. For example, colibactin-associated mutations may pre-dispose individuals to cancer development but require successive microbial “hits” to fully realize a malignant consequence. Such a mechanism may explain the increased risk of CRC in patients colonized with *pks^+^* bacteria that adhere to a high-fat Western diet, or the co-occurrence of *pks^+^ E. coli* and ETBF in CRC patients with hereditary APC mutations and the observation that increased tumorigenesis after ETBF + *pks^+^ E. coli* dual association in *Apc^Min/+^* mice can be abrogated by the deletion of either *bft* or *pks* genes, respectively.^23^ The recent identification of multiple genotoxins encoded by commensal bacteria isolated from IBD patients^18^ suggests that there may be a plethora of similar interactions that remain to be discovered. Thus, developing an accurate assessment of the role for bacterial drivers of CRC will likely require incorporating proteomic, genomic, and transcriptomic data capable of holistically anatomizing complex microbial ecosystems. Several technological advances may facilitate the acquisition of knowledge that can more directly answer these questions. Using spatial bacterial transcriptomics and transcriptional recording techniques, researchers can begin probing the effects of heterogenous microbial communities on host gene expression at the single-cell level^187^ or how transient changes in the intestinal environment may influence microbial gene expression and alter carcinogenic potential.^188^ Moreover, the advent of novel *in situ* metabolomics may allow scientists to directly study the interaction of microbial metabolites with immune cells or pre-cancerous lesions.^189^ This complexity is compounded by cancer progression itself, during which tumor heterogeneity is influenced by a unique “intra-tumoral microbiome”. In such cases, intracellular bacteria modulate gene expression^187^ and antigen presentation^190^ in ways that may enhance malignant transformation. Microbial ecosystems in the gut also contain extensive fungal and viral communities that can promote CRC development.^191–195^ While significant advances have been made, cancer microbiome research is a nascent field. The hope of cancer microbiome research is that unraveling these complexities may lead to a better understanding of the factors influencing an individual patient’s disease progression, and thus lead to better risk-assessment or treatment.

How can one leverage this knowledge to improve CRC outcomes? One possibility is to “edit” the intestinal bacteriome by the targeted removal of carcinogenic bacterial species using bacteriophages. Pre-clinical studies suggest phage-targeting of *pks^+^ E. coli* can reduce their oncogenic effects *in vivo*.^196^ Such an approach may be feasible in humans using species-specific phage cocktails, as demonstrated by early-phase clinical trials targeting *K. pneumoniae* in a small cohort of healthy patients.^197^ Alternatively, the biochemical characterization of microbial metabolic pathways involved in promoting CRC may allow for the development of small molecule inhibitors directly inhibiting these pathways.^198,199^ On the other hand, the addition of bacteria with preventative or treatment-enhancing effects may be administered to counteract cancer progression and boost treatment efficacy. A more complete mechanistic understanding of these effects may allow for the engineering of microbes that produce specific antigens or metabolites that activate anti-tumor immunity or apoptotic pathways in cancer cells.^6,200,201^ In cases where microbial metabolism interferes with therapy by reducing efficacy or exacerbating dose-limiting side effects, synthetic inhibitors may be used to interrupt these biosynthetic pathways.^202^ In theory microbial colonization may be bypassed altogether, and cell-free reactive microbial metabolites administered as treatment or therapeutic adjuvants.^185^ Collectively, the microbiome holds great promise as a source of individual variability that may help explain diverse patient outcomes. Future clinical approaches may utilize this knowledge to understand the root cause of tumor development in CRC on a patient-by-patient basis, or to help inform therapeutic approaches.

## Data Availability

No new data were generated or analyzed in support of this research.
